# Reproductive outcome of septate uterus following hysteroscopic septum resection

**DOI:** 10.4103/0974-1208.74157

**Published:** 2010

**Authors:** Priya Selvaraj, Kamala Selvaraj

**Affiliations:** Fertility Research Centre, G G Hospital, 6E - Nungambakkam High Road, Chennai - 600 034, India

**Keywords:** BOH, hysteroscopy, infertility, recurrent pregnancy loss, septate uterus, septum resection

## Abstract

**OBJECTIVE:**

To evaluate the reproductive outcome following hysteroscopic septum resection in patients with primary and secondary (recurrent pregnancy loss [RPL] and bad obstetric history [BOH]) infertility.

**STUDY DESIGN:**

Retrospective study.

**MATERIALS AND METHODS:**

Hysteroscopic septum resection was performed on 26 patients with a history of either recurrent pregnancy loss, BOH or infertility. The septum resection was performed using a bipolar versapoint system. Reproductive performance of these patients after septum resection was analyzed. The main outcome measures were clinical pregnancy and live birth rates.

**RESULTS:**

Hysteroscopic septum resection was performed on seven patients with the history of secondary infertility. Post operatively, the pregnancy rate was 86% (*n*=6), and the live birth rate was 67% (*n*=4). After septum resection in 19 primary infertile patients, 6 (32%) patients conceived which resulted in live birth rates of 67% (*n*=4).

**CONCLUSION:**

Hysteroscopic septum resection using bipolar versapoint system is an effective and safe approach for the removal of septum. Hysteroscopic septum resection in women with septate uterus significantly improves the live birth rates and future fertility is not impaired.

## INTRODUCTION

Incomplete resorption of the mullerian duct during embryogenesis leads to mullerian anomalies which may alter the reproductive outcome of the patients. The mean incidence of uterine defects in the general population and in infertile women is 4.3%.[1] In patients with recurrent pregnancy loss, the incidence of uterine defects increases by 5-25%.[2,3] Septate uterus (class V, American fertility classification[4]) is the most common congenital uterine anomaly, comprising approximately 55% of mullerian duct anomalies.[5]

A septate uterus is not a primary factor for infertility.[6] Nearly 40% of patients with septate uterus have reproductive failure, obstetrical complications and an increased incidence of recurrent miscarriages.[7] Clinically, symptoms may range from being asymptomatic thus remaining undiagnosed, to the development of poor reproductive outcome.[8]

Incidental discovery of a uterine septum may sometimes occur during the evaluation of infertility. Uterine septum resection by a hysterolaparoscopic approach has been found to be beneficial with significant improvement in pregnancy rates post procedure. This has many advantages such as shorter operating and hospitalization periods, reduced risk of post operative pelvic adhesions, low morbidity and an increased rate of vaginal delivery.

This retrospective study has been designed to evaluate the impact of hysteroscopic septum resection on the reproductive outcome of patients with a history of primary and secondary infertility.

## MATERIALS AND METHODS

The reproductive efficiency in 26 patients with septate uterus who underwent hysteroscopic septum resection in our fertility research center from January 2006 to December 2008 was analyzed. Since we focused only on septate uterus in our study, we excluded the patients with associated genital or pelvic diseases (myomas, endometriosis, adhesions and pelvic inflammatory disease). Women aged > 35 years were also excluded in order to avoid the chronological age related infertility. Any other known cause of infertility was not included in this study.

The presenting complaints in these patients included secondary infertility (7/26, 27%) and unexplained infertility (19/26, 73%). Of the patients with secondary infertility (*n*=7), 4 (RPL) of them had three to four previous first trimester pregnancy losses (83%) while the remaining three (BOH) had a previous cesarean section for preterm labor (at less than 28 weeks gestational age) which resulted in neonatal mortality.

In our center, the gold standard for diagnosing uterine anomalies has always been hysterolaparoscopy. The advantage of combining laparoscopy with hysteroscopy is well known. It offers a dual advantage of assessing the pelvis as well as the uterine cavity, aiding diagnosis and correlating or confirming findings crucial to therapy in cases of uterine anomalies. If a suspicion is elicited in history or ultrasound of a septate uterus, this procedure which is considered as a gold standard in evaluation of infertile couples, enables us to operate and resect the septum in a single sitting. If a clear, pre procedure awareness is created and a consent obtained for performance of the surgery, it avoids a second look and operative hysteroscopy, saving time and finances for the couple.

Under general anesthesia, hysteroscopic septum resection was performed using unique bipolar versapoint system (Gynecare, Johnson and Johnson, Somerville, New Jersey) with normal saline as the distension medium to avoid potential hyponatremia.[9,10] Power settings ranged from 50 W to 200 W. The versapoint system offers three bipolar electrode tip configurations – twizzle electrode for vaporization and needle-like cutting, spring and ball electrode for rapid and precise tissue vaporization and desiccation, respectively.

After assessing the direction and length of uterocervical canal, the cervix was dilated using Mathew Duncan dilators up to size 15. The operative external sheath of the versapoint of 9 mm diameter was introduced and hysteroscopy was performed. Versapoint spring electrode was assembled and the septum was divided into and fro upward direction until complete visualization of both tubal ostia. There were no complications such as uterine perforation, excessive bleeding, fluid overload or thermal injury. Post procedure, all the patients were put on hormone replacement for one to two cycles to enable recovery of endometrium. The endometrium was prepared with incremental doses of estrogen (Estradiol Valerate 2 mg, German Remedies) commencing from day 2 or day 3 of the cycle to a maximum of 8mg/day with initiation of uterogestan (natural micronized progesterone, Solvay Pharma India ltd) 200 mg thrice a day. They were subsequently treated by assisted reproductive technology (ART) or advised for natural conception. A transvaginal ultrasonography performed at 38 days in cases with a positive β - hCG result confirmed intrauterine pregnancy.

### Statistical analysis

The statistical analysis of the data was performed using SPSS version 14.0 Software (Chicago, IL, USA). Continuous variables are presented as mean and SD and were analyzed by independent *t*-test. Categorical data are represented by frequency with percentage and were analyzed by chi-square and Fischer exact test. In data analysis, *P*< 0.05 was considered as statistically significant.

## RESULTS

All septum were successfully resected with no hysteroscopic or anesthetic complications. Demographic characteristics of the patients at the time of surgery and the initial pregnancy outcome following septum resection are shown in Table 1. 7 patients had hysteroscopic septum resection in secondary infertility group and 6 patients achieved pregnancy post procedure (86%). Of the 6 patients who conceived, 2 had a first trimester pregnancy loss (33%) in RPL group and 4 (67%) had live births at term by elective cesarean. In the infertility group, 6 (32%) patients conceived after septum resection. Of the 6 pregnancies, 2 (33%) resulted in a first trimester loss and 4 (67%) resulted in live births. Following hysteroscopic septum resection, the spontaneous abortion rate in patients with secondary infertility decreased from 83% to 33% (*P*=.021 statistically significant) and live birth rate was increased to 67%.

**Table 1:** Demographic characteristics and reproductive outcome after septum resection

Parameters	Primary infertility (%)	Secondary infertility (%)
No. of patients	19	07
Age (years) Mean ± SD	31.50 ± 3.02	27.50 ± 2.95
Duration of follow-up (Months) Mean ± SD	10.33 ± 6.47	9.83 ± 6.18
Pregnancies (n)	06 (31.6)	06 (85.7)
Natural (n)	01 (16.7)	02 (33.3)
ART (n)	05 (83.3)	04 (66.7)
Miscarriages (n)	02 (33.3)	02 (33.3)
Term deliveries (n)	04 (67)	04 (67)

## DISCUSSION

The etiology of infertile patients with uterine anomalies remains unclear. The mechanisms of septate uterus causing early pregnancy loss and infertility have not been established.[11] In our patients, the septum was identified during infertility evaluation. We confirmed the diagnosis by performing diagnostic hysterolaparoscopy which enables us to operate and resect the septum at the same sitting. Few studies also have stated that diagnostic hysteroslaparoscopy is now accepted as the most effective approach.[1,11] Our study supports the use of hysteroscopic septum resection to improve pregnancy outcome in patients with a history of recurrent pregnancy loss (86%) and infertility (32%). Szymansky *et al*., reported that women with infertility after septum resection have an increased pregnancy rate.[10] A hysteroscopic guided septum resection not only eliminates an unsuitable site for implantation but also results in a better endometrial function, probably through re-vascularization of the connective tissue of the uterine fundus and significantly improves nidation.[12]

The septum is thought to be composed of fibroelastic tissue with inadequate vascularization and altered relations between myometrial and endometrial vessels, thus exerting a negative effect on fetal placentation.[13] However, our findings are similar to those identified in a review of reproductive outcome before and after hysteroscopic septum resection. In a literature survey, miscarriage and preterm delivery rates prior to septum resection were 88% and 9% respectively, and the live birth rate was only 3%. After septum resection, these rates were 14%, 6% and 80% respectively.[14,15]

Grimbizis *et al*., retrospectively examined the reproductive performance of infertile patients before and after septum resection and found that septum resection does not impair fertility, both in sub-septate and complete septum post procedure.[1] Our study also showed that hysteroscopic septum resection did not impair future fertility in cases of complete septum.

We initiated treatment cycles two months post procedure in contrast to a larger study by Kormanyos *et al*.,[11] where treatment cycles were carried out after a minimum of four months. However, this interval between procedure and treatment cycle did not alter the pregnancy outcome. Although our series were smaller in comparison, the pregnancy rates were significantly higher following septum resection. Although the incidence is rare, there have been case reports of rupture post hysteroscopic septum resection in recent years.[16] In our study, there have been no cases of post operative uterine rupture which is one of the most serious complications or other complications related to procedure such as fluid and electrolyte imbalance, thermal injury or perforation.

## CONCLUSION

Hysteroscopic septum resection is a safe and efficacious procedure. The treatment is beneficial for high risk pregnancies such as recurrent pregnancy loss and infertility. Moreover, because of its simplicity, minimal invasiveness and low morbidity, it is considered as a liberal approach to the treatment of polyps, intracavity fibroids as well as uterine septum. Last but not least; our study concluded that septum is the major cause of pregnancy loss.
